# Epistemic conflict in neurodevelopmental assessment: a critical discourse analysis of second opinion requests

**DOI:** 10.3389/fpsyg.2026.1832985

**Published:** 2026-07-15

**Authors:** Marios Adamou, Bethany Clayton, Niki Kyriakidou

**Affiliations:** 1 School of Human and Health Sciences, University of Huddersfield, Huddersfield, United Kingdom; 2 South West Yorkshire Partnership NHS Foundation Trust, Wakefield, United Kingdom; 3 Leeds Beckett University, Leeds, United Kingdom

**Keywords:** ADHD, autism, critical discourse analysis, diagnostic disagreement, epistemic injustice, gender bias

## Abstract

**Background:**

Adult ADHD and autism diagnostic services in the National Health Service (NHS) face severe capacity constraints. When assessments conclude that diagnostic criteria are not met, some individuals contest these decisions by requesting a formal second opinion.

**Objective:**

This service evaluation examined how patients linguistically construct challenges to clinical diagnostic decisions in second opinion requests following negative ADHD or autism assessments.

**Methods:**

We analysed 51 written second opinion requests submitted to an NHS adult neurodevelopmental diagnostic service using Fairclough’s critical discourse analysis framework, examining linguistic features, discursive practices, and social contexts. Demographic data were analysed using descriptive statistics; inferential tests were accompanied by effect sizes and an explicit power note. A trustworthiness framework comprising analytic memos, conservative coding, negative case analysis, peer debriefing within the interdisciplinary team, and an audit trail substituted for inter-rater reliability where a second coder was not feasible.

**Results:**

The sample consisted of predominantly women (68.6%) with a mean age of 36.7 years. Four discursive strategies emerged: appropriation and recalibration of clinical taxonomy (98.0%), mobilization of biographical and pharmacological evidence (66.7%), hermeneutic reframing of clinical benevolence (98.0%), and temporal conflict of masking (58.8%). A fifth phenomenon, the competence paradox (86.3%), is reframed as a structural-discursive bind imposed by the genre rather than a patient strategy. The near-universality of each strategy individually, rather than any non-independent co-occurrence, characterized the rhetorical pattern. Inferential analyses on the self-referred subgroup (*n* = 39) were underpowered: only effects of d > 1.0 were detectable. Reported effect sizes were small (Cramer’s *V* = 0.20; Cohen’s *d* = 0.12 for age by condition; *d* = 0.45 for age by gender), with confidence intervals consistent with both null and substantial effects.

**Conclusion:**

Second opinion requests reveal three forms of epistemic injustice: testimonial (experiential knowledge discounted), hermeneutical (inadequate medical terminology), and a distinct procedural form (challenge genres systematically filter out those whose impairment makes genre-competent performance impossible and paradoxically render genre-competent performance as counter-evidence). These findings underscore the need for assessment pathways that prospectively capture masking, compensation, functional cost, and biographical impairment, supported by transparent diagnostic documentation, targeted assessor training, and accessible routes for reconsideration that reduce the competence paradox.

## 1 Introduction

### 1.1 The crisis in NHS neurodevelopmental diagnostic services

Adult neurodevelopmental diagnostic services in the United Kingdom NHS face unprecedented demand alongside severe capacity constraints (Smith et al., 2024). Waiting times for ADHD and autism assessment routinely exceed 2 years, with some services reporting waits approaching 5 years (NHS Digital, 2024). This capacity crisis coincides with documented increases in referral rates, particularly amongst women and people seeking late diagnosis after years or decades of unrecognized difficulties (Knott et al., 2024). Within the NHS context of resource scarcity and escalating demand, diagnostic decisions carry substantial consequences, as they determine eligibility for the workplace accommodations, educational support, pharmacological treatment, and psychological therapies that the National Institute for Health and Care Excellence guidance for ADHD (National Institute for Health and Care Excellence, 2018) and autism (National Institute for Health and Care Excellence, 2012) makes contingent on a confirmed diagnosis, together with the explanatory frameworks that enable people to understand lifelong patterns of difficulty.

Diagnostic disagreement within this NHS service context becomes likely when outcomes fail to meet expectations. When the assessment concludes that the criteria are not met, whether through the absence of sufficient symptoms, lack of functional impairment, attribution to alternative explanations, or threshold judgments about severity, some people experience this outcome as invalidating. Years of waiting culminate in the denial of recognition for difficulties they have experienced as pervasive and debilitating. For many, particularly women and those whose academic, occupational, or social performance remains superficially intact through effortful compensation (sometimes labeled high-functioning) (Lai et al., 2017; Loomes et al., 2017; Young et al., 2020), adverse outcomes are perceived to reflect systematic bias within diagnostic frameworks normed predominantly on male, overtly impaired presentations (Cruz et al., 2025; Srivarathan et al., 2025).

In this context, some patients respond to negative diagnostic outcomes by submitting formal second opinion requests, challenging clinical decisions through institutional procedures. These requests constitute a particular genre of patient-clinician communication wherein asymmetric power relations become explicitly contested. Unlike routine medical interactions in which professional authority remains largely unquestioned, second opinion requests directly challenge clinical expertise, asserting that the diagnostic determination was inaccurate and should be reconsidered.

### 1.2 Neurodevelopmental diagnosis as epistemic conflict

Neurodevelopmental diagnosis represents a contested site of epistemic conflict for several reasons. First, unlike many medical conditions diagnosable through objective biomarkers, ADHD and autism diagnosis relies substantially on subjective symptom reporting, behavioral observation, and clinical judgment about whether the presentation meets threshold criteria (American Psychiatric Association, 2013; World Health Organization, 2019). There is no blood test, brain scan, or genetic marker that definitively confirms or excludes diagnosis. The clinical assessment therefore depends on three sequential steps within the diagnostic process (Adamou et al., 2024): (i) patients accurately reporting their internal experiences, (ii) clinicians reliably eliciting and describing behavior patterns, and (iii) clinicians correctly interpreting that information within standardized frameworks. Each of these three steps introduces interpretive flexibility and scope for disagreement (Kraemer, 2007).

Second, the diagnostic criteria themselves involve substantial ambiguity. DSM-5 (American Psychiatric Association, 2013) and ICD-11 (World Health Organization, 2019) specify symptom clusters, age-of-onset requirements, functional impairment thresholds, and exclusion criteria, but application to specific individuals requires judgment. How severe must inattention be to constitute clinically significant impairment? How pervasive must social difficulties be to meet autism criteria? When do compensation strategies indicate the absence of impairment versus evidence of necessary adaptation? These questions lack definitive answers, creating space for reasonable disagreement between patients and clinicians whilst enabling threshold modulation responsive to non-clinical pressures, including resource availability.

Third, the heterogeneity of presentation within diagnostic categories creates further ambiguity. ADHD and autism presentations range from severe, obvious, and pervasive difficulties to subtle, context-dependent, and partially compensated patterns. Women, high-IQ individuals, and those who develop sophisticated compensation strategies may meet diagnostic criteria whilst appearing superficially functional during brief assessment (Lai et al., 2017). Clinical frameworks struggle to accommodate this heterogeneity, often defaulting to prototypical presentations whilst pathways to diagnosis narrow for atypical or well-compensated individuals.

Finally, the length of the assessment period has inherent limitations. Standard psychiatric history-taking involves relatively short time spent with patients, typically several hours across one or two sessions, during which clinicians observe behavior, elicit symptom reports, and review historical information (Adamou et al., 2024). Even with collateral information, a single assessment can miss long-term patterns, situational variability, and the accumulating burden of compensating strategies. Patients may push themselves to perform during high-stakes testing but still struggle significantly in everyday contexts.

### 1.3 Aims and research questions

Against this background, the present service evaluation employs critical discourse analysis (CDA) to examine how patients linguistically construct challenges to clinical authority within second opinion requests. We address three research questions:

RQ1. What discursive strategies do patients deploy to construct counter-authority against clinical decisions? This question examines micro-level linguistic choices including vocabulary selection, grammatical structures, modality markers, and rhetorical organization in the sense developed within established traditions of rhetorical analysis (Leach, 2000).

RQ2. How do these strategies draw upon and transform available discourse types? This question examines the discursive practice dimension, analysing intertextuality, interdiscursivity, and genre transformation.

RQ3. What do these linguistic strategies, and the structural-discursive binds they encounter, reveal about power relations, epistemic hierarchies, and procedural constraints within neurodevelopmental diagnostic contexts?

## 2 Literature review and theoretical framework

The study draws together three previously separate literatures which are CDA in healthcare encounters, epistemic injustice in clinical contexts, and the contested area of adult neurodevelopmental diagnosis. Each trajectory is reviewed from foundational to recent contributions, and each closes with the specific question the present corpus is uniquely positioned to address.

### 2.1 Critical discourse analysis in healthcare encounters

Fairclough’s three-dimensional model of discourse (Fairclough, 1992, 2013) treats language as simultaneously a text, a discursive practice, and a social practice. The model has been operationalized across healthcare settings to expose how clinical authority is enacted linguistically and how patients respond. Ainsworth-Vaughn’s (Ainsworth-Vaughn, 1998) analyses of physician-patient consultations demonstrated that the doctor-patient asymmetry is sustained through topic control, question design, and the differential use of technical lexis. Iedema’s (2007) work on the textualization of clinical reasoning showed how nominalization and passive constructions convert provisional interpretations into apparently settled facts, occluding clinician agency. Wodak’s (2009) Discourse-Historical Approach extended CDA by formalizing the analysis of strategies, those systematic ways of using language to achieve social, political, or interactional ends, providing the conceptual scaffolding on which the present analysis builds. Sarangi and Roberts (1999) applied CDA to multi-party clinical interactions, distinguishing institutional from professional and personal discourse orders, and showed how patient accounts must be translated into institutional categories to acquire uptake. Candlin and Candlin (2002) mapped how genre conventions in clinical correspondence constrain what can be said and by whom.

Within this trajectory, attention has progressively shifted from face-to-face consultation to written and institutional genres. Patient resistance through discourse (Putnam et al., 2005) has been documented in oral consultation and in informal narrative, but the written challenge of institutional process, formal correspondence submitted after a diagnostic non-confirmation and intended to reopen a closed clinical decision, has not been systematically analysed. This is the gap the present study addresses: CDA of written institutional challenges following diagnostic non-confirmation in adult neurodevelopmental services.

### 2.2 Epistemic injustice in clinical contexts

Fricker’s (2007) account of epistemic injustice identifies two species. Testimonial injustice takes place when a hearer assigns a deflated level of credibility to a speaker’s word owing to prejudice; hermeneutical injustice occurs when a gap in collective interpretive resources places someone at an unfair disadvantage when making sense of their social experience. Carel and Kidd (2014) extended Fricker’s framework to medicine, arguing that ill patients are systematically positioned as epistemically deficient because their first-person accounts are taken as expressive rather than informational. Wardrope (2015) examined how diagnostic categories themselves may instantiate hermeneutical injustice when they impose ill-fitting interpretive frames. Crichton et al. (2017) mapped epistemic injustice across mental healthcare, including its compounding effects in long-term psychiatric care. Kurs and Grinshpoon (2018) applied the framework to involuntary psychiatric treatment, demonstrating how procedural mechanisms can entrench rather than redress epistemic harm. Dotson (2011) describes contributory injustice as a situation where someone is not properly heard because the listener refuses, or fails, to use the interpretive tools needed to understand them.

Procedural barriers to challenging clinical decisions are widely noted, but no study has theorized how the genre requirements of institutional challenge procedures, the formal, written, evidence-rich, criterion-aligned text demanded of a complainant, themselves constitute a distinct form of epistemic injustice. This is the second gap the present study addresses: a theoretical articulation of procedural epistemic injustice, distinguished from access barriers, from testimonial discounting, and from contributory injustice.

### 2.3 Neurodevelopmental diagnostic disputes

Adult ADHD and autism diagnosis sits within a contested empirical and political landscape. The male preponderance historically reported in autism prevalence has been challenged by recent meta-analytic work; Loomes et al. (2017) estimated a true sex ratio closer to three to one rather than the four-to-one figure of earlier prevalence studies, attributing the gap to diagnostic under-ascertainment in females. The expert consensus statement of Young et al. (2020) documented systematic underdiagnosis of ADHD in girls and women, attributable to symptom profiles dominated by inattentive rather than hyperactive features, internalizing comorbidity, and gendered socialization pressures that promote compensatory behavior. Lai et al. (2017) quantified camouflaging (also termed masking) in autistic adults, suggesting that effortful social compensation is associated with mental health burden and with delayed or missed diagnosis. Cruz et al. (2025) put forward a persistent male-leaning diagnostic bias in autism through systematic review and meta-analysis. Srivarathan et al. (2025) highlighted diagnostic inequities at the intersection of autism and minoritized demographic positions.

Parallel work has examined the role of online neurodiversity communities as sites of alternative knowledge production. Chapman (2024) situates the neurodiversity movement within a broader politics of mental health, identifying community-generated terminology (time blindness, task paralysis, autistic burnout) as collective interpretive resources that fill conceptual gaps left by clinical nosology. However, there has been little systematic analysis of how patients use these shared interpretive resources in written complaints to challenge a clinical decision. This is the third gap addressed by the present study. It examines how patients draw on clinical knowledge, community-based understandings, and personal experience within a high-stakes written format, and how these patterns relate to demographic differences in diagnostic disputes.

### 2.4 Integrating the three trajectories

Bringing CDA, epistemic injustice theory, and neurodevelopmental diagnostic research together allows the issue to be understood at three levels. At the textual level, CDA shows the language choices patients use. At the discursive practice level, it shows how patients combine clinical language, community language, and personal experience. At the social practice level, epistemic injustice theory helps explain what these combinations show about institutional power, restrictive complaint formats, and the conditions under which patients’ accounts of neurodevelopmental impairment are either recognized or dismissed. Together, these approaches provide the analytical basis for this study.

## 3 Methodology

### 3.1 Design

The work is a service evaluation employing CDA on a complete corpus of second opinion requests submitted to a single NHS adult neurodevelopmental diagnostic service across a defined 12-month period. The design is qualitative, interpretive, and theory-driven, with quantitative descriptive and inferential components reporting demographic associations.

### 3.2 Data collection and governance

All written second opinion requests submitted between 1 January 2025 and 31 December 2025 requesting reconsideration of a diagnostic decision were included. The service evaluation was registered with the Trust’s clinical audit and service evaluation function before extraction. All identifying information was removed at extraction; exemplars quoted in the paper have been further redacted (specific numerals altered, occupational descriptors generalized) so that no patient is identifiable.

We acknowledge the ethical complexity of analysing texts produced under vulnerable circumstances by patients contesting decisions they experience as profoundly invalidating. We treat subjective distress as legitimate regardless of diagnostic accuracy, maintain analytical neutrality regarding clinical correctness, avoid dismissive characterization, and remain transparent about analytical limitations and the effects of researcher positioning.

### 3.3 Researcher positionality and reflexivity

The study was conducted by an interdisciplinary team consisting of a clinician (MA), a senior NHS administrator, and a human resources academic. The clinician contributed expertise in neurodevelopmental assessment, the administrator brought knowledge of NHS systems, and the human resources academic supported analysis of organizational power and equity, including gender-related issues.

The team met every 2 weeks during the analysis period to review and discuss emerging findings. Minutes from these meetings were kept as part of the audit trail.

### 3.4 Corpus and demographic analysis

The corpus consisted of 51 written second opinion requests. For each request, we extracted gender, age at submission, condition (ADHD, autism), and referral source (self-referral, third-party). Descriptive statistics were calculated for the complete corpus. To minimize selection effects arising from externally initiated referrals, inferential analyses were restricted to the self-referred subgroup (*n* = 39). Fisher’s Exact Test examined the association between gender and condition; independent samples *t*-tests examined age differences. Following recent guidance on reporting under small samples (Cohen, 1988), every inferential test is accompanied by an effect size (Cramer’s V for the contingency analysis; Cohen’s d with 95% confidence interval for the *t*-tests) and by an explicit power note. The minimum detectable effect at alpha = 0.05 and power = 0.80 across the available cell sizes was Cohen’s d of approximately 1.0; consequently, only very large effects would be reliably detected.

### 3.5 Fairclough’s three-dimensional framework

Analysis followed Fairclough’s three-dimensional framework (Fairclough, 1992, 2013), applied via a systematic four-stage coding process in NVivo 14.

Dimension 1: Textual Analysis (Micro-level). We analysed lexis (technical medical terms versus lay descriptions), transitivity (how agency was attributed, for example The assessment failed to capture my symptoms versus I feel you missed this), and modality (expressions of certainty, for example I definitely meet the criteria versus I feel I might have this condition).

Dimension 2: Discursive Practice (Meso-level). We examined production and distribution and how texts interact with other genres, including intertextuality (how patients quoted assessment reports, DSM-5, or online resources) and genre mixing (how requests blended medical history with legal complaint conventions).

Dimension 3: Social Practice (Macro-level). We linked specific linguistic features to wider power dynamics, including doctor-patient hierarchies, resource scarcity, threshold-based rationing, and gendered assumptions about credibility. Following peer review, social practice interpretation is presented in the Discussion rather than embedded in Results.

### 3.6 Identifying discursive strategies and structural tensions

We define a discursive strategy as a goal-directed, recurrent configuration of linguistic choices that performs a recognizable institutional action within a specific genre. Strategies sit analytically between micro-level features (lexis, modality, transitivity) and macro-level structures (institutional power, evidential hierarchies). This conceptualization aligns with CDA accounts that treat discourse as socially constitutive (Fairclough, 1992, 2013) and with Wodak’s (2009) Discourse-Historical Approach.

We distinguish strategies (deployed by patients to achieve interactional ends) from structural-discursive tensions, which are emergent features produced by the genre itself rather than by patient choice. The fifth phenomenon we report, the competence paradox, is reclassified in this revision as a structural-discursive tension because it is built into the nature of second opinion requests rather than chosen by the patient. Section “5 Discussion” explicates the analytic and theoretical implications.

Discursive strategies emerged through a systematic four-stage analytical process. Stage 1 involved sequential corpus reading and the generation of analytic memos, capturing repeated rhetorical moves, lexical clusters, stance patterns, and evidential warrants across the 51 requests. Stage 2 employed systematic coding, marking textual features including nominalization patterns, technical noun clusters, modality markers, evidential quantification, reported speech, contrastive framing, temporal operators, causal constructions, and meta-commentary on assessment processes. Stage 3 grouped co-occurring features into functions when they recurred across documents and performed the same social action; NVivo matrix coding queries enabled systematic examination of co-occurrence patterns. Stage 4 stress-tested provisional clusters against the complete corpus, retaining only strategies that met three simultaneous criteria: recurrence across multiple texts, internal coherence with identifiable linguistic indicators, and explanatory value with respect to the social practice level.

Strategies were coded for presence or absence within each second opinion request rather than for frequency of occurrence. A strategy was coded present if at least one qualifying segment occurred anywhere in the document. A segment qualified when two conditions were met: (i) at least two diagnostic markers co-occurred within the same local passage (sentence cluster or paragraph), and (ii) the markers converged on the strategy’s defining function. A strategy was not coded when only isolated terminology appeared without the functional move, or when another strategy better explained the move. Where ambiguity existed, the conservative rule applied: explicit linguistic convergence was required rather than inferring intent from isolated features.

### 3.7 Trustworthiness in place of inter-rater reliability

Coding was undertaken by a single researcher (MA). We acknowledge that this is a constraint on the analysis and that, for an interpretive methodology making claims about recurrent discursive strategies and their social-structural significance, a bare reflexivity statement is insufficient. Recruitment of a second coder for a 20 percent subsample was considered but proved infeasible within the time and resource constraints of the service evaluation. We therefore substituted a defensible trustworthiness framework grounded in interpretive research traditions (Lincoln and Guba, 1985; Nowell et al., 2017; Braun and Clarke, 2023), comprising four components.

First, analytic memos were written after each reading of each request, capturing initial impressions, deviant features, and developing interpretive hypotheses; these were later cross-referenced with the final coding scheme.

Second, the conservative coding rule (two convergent markers within a local passage, both pointing to the same defining function) systematically disfavoured over-coding. Where a single feature could plausibly index more than one strategy, the more cautious classification was applied.

Third, negative case analysis was performed at Stage 4: provisional clusters were tested against requests in which they were absent or attenuated, and the coding scheme was revised until each remaining strategy survived examination against potentially disconfirming texts. Two preliminary strategies that did not survive this step (an explicit-rights strategy and an external-validation strategy) were abandoned.

Fourth, exemplar quotation density: every claim about a discursive strategy is anchored to specific textual exemplars in section “4 Results,” enabling readers to assess the warrant for each interpretation against the underlying data.

We make no claim that these procedures fully substitute for inter-rater reliability quantification. They are offered as the strongest available defensible alternative within the methodological tradition of interpretive CDA, and we discuss their residual limitation in section “7 Limitations.”

## 4 Results

### 4.1 Demographic characteristics

Table 1 summarizes the demographic and clinical characteristics of the full cohort (*N* = 51). The majority were female (35 of 51 individuals, 68.6%); the remaining 16 (31.4%) were male, with the two values summing to the full cohort. The mean age was 36.7 years (SD 12.1; range 19–63 years). The primary reason for seeking a second opinion was ADHD (36 of 51, 70.6%); the remaining 15 (29.4%) concerned autism, the two values again summing to the full cohort. Most referrals were initiated by the patients themselves (39 of 51, 76.5%); the remaining 12 (23.5%) came from third parties, including family members and General Practitioners. All percentages are expressed to one decimal place and are derived from the absolute counts shown.

**TABLE 1:** Baseline characteristics of the study population (*N* = 51).

Variable	Category	*n*	%
Gender	Female	35	68.6
	Male	16	31.4
Condition	ADHD	36	70.6
	Autism	15	29.4
Referral source	Self-referral	39	76.5
	Third-party	12	23.5
Age (years)	Mean (SD)	36.7 (12.1)	–
	Range	19–63	–

### 4.2 Inferential analyses in the self-referred subgroup (*n* = 39)

To minimize potential selection bias from externally initiated referrals, inferential analyses were restricted to the 39 individuals who self-referred. Within this subgroup, 28 participants were female and 11 male. A Fisher’s Exact Test examined the association between gender and condition: no statistically significant association was identified, *p* = 0.262. The corresponding effect size was small (Cramer’s *V* = 0.20). A higher proportion of male applicants sought an autism assessment (5 of 11, 45.5%) compared with female applicants (7 of 28, 25.0%); 75.0% of female applicants requested an ADHD assessment, compared with 54.5% of male applicants. Independent samples *t*-tests examined age differences: no significant difference in mean age between those seeking a second opinion for ADHD (*M* = 38.1, SD = 12.5) and those seeking one for autism (*M* = 36.7, SD = 11.2), t(37) = 0.33, *p* = 0.743, Cohen’s *d* = 0.12, 95% CI [−0.57, 0.80]; no significant difference between female (*M* = 39.1) and male (*M* = 33.9) applicants, t(37) = 1.25, *p* = 0.220, Cohen’s *d* = 0.45, 95% CI [−0.26, 1.15].

We note that these inferential tests are severely underpowered. With cell sizes of 11–28, the minimum detectable effect at alpha = 0.05 and power = 0.80 was Cohen’s d of approximately 1.00; only very large effects would be reliably detected. The non-significant results should not be interpreted as evidence of absence. The effect sizes obtained for age by gender (*d* = 0.45) and the contingency analysis (*V* = 0.20) are consistent with the possibility of small to moderate true effects that this corpus is insufficient to confirm or exclude. We therefore treat the inferential results as descriptive rather than confirmatory and base substantive inference on the descriptive demographic distribution.

### 4.3 Discursive strategies: Prevalence and patterns

Systematic four-stage coding identified four discursive strategies (S1-S4) and one structural-discursive tension (T1, the competence paradox). The classification of T1 as a tension rather than a strategy is theoretically motivated and is explained in section “5 Discussion.” Table 2 reports prevalence across the 51 requests.

**TABLE 2:** Prevalence of patient strategies (S1–S4) and the structural-discursive tension (T1) across 51 requests.

Code	Phenomenon	Type	*n*	%
S1	Appropriation and recalibration of clinical taxonomy	Strategy	50	98.0
S2	Mobilization of biographical and pharmacological evidence	Strategy	34	66.7
S3	Hermeneutic reframing of clinical benevolence	Strategy	50	98.0
S4	Temporal conflict of masking	Strategy	30	58.8
T1	Competence paradox	Structural-discursive tension	44	86.3

Two strategies were near-universal: S1 (98.0%) and S3 (98.0%). T1 was highly prevalent (86.3%). Two strategies showed moderate variation: S2 (66.7%) and S4 (58.8%). Requests rarely deployed strategies in isolation. The mean number of patient strategies per request was 3.22 of a possible four (SD 0.78); 76.5% deployed three or more strategies simultaneously (Table 3).

**TABLE 3:** Distribution of strategy combinations across 51 requests.

Number of patient strategies (of 4)	*n*	%
1	2	3.9
2	9	17.6
3	20	39.2
4	20	39.2

The important distributional feature is the near-universality of each of S1 and S3 individually (98.0% each). Their joint occurrence does not constitute an independent finding: under statistical independence, two strategies each occurring in 98.0% of requests would be expected to co-occur in approximately 0.98 × 0.98 = 96.04% of requests by chance alone. The observed co-occurrence (96.1%) is therefore not informative beyond the marginal prevalence of each strategy and is not reported as a separate result.

S2 (biographical and pharmacological evidence) appeared more frequently in ADHD requests (72.7%) than in autism requests (55.6%). S4 (temporal conflict of masking) varied markedly by author type: professional advocates deployed it universally (100%), whereas patients writing alone did so in 42.9% of cases. We defer interpretation of these patterns to the Discussion.

### 4.4 Strategy 1: appropriation and recalibration of clinical taxonomy (98.0%)

This strategy involved substituting everyday descriptions with diagnostic nomenclature through nominalization, while recalibrating implied diagnostic thresholds. Outward functioning was textually positioned as inadequate to disconfirm the claim of impairment because impairment was construed as internal cost, delayed collapse, or uneven performance across contexts.

Exemplar from a woman in her forties:

*The impression I got after the conversation with Dr XXX is that a life with ADHD should be filled with problems at school and failure at work… Many people diagnosed with ADHD can function OK in their daily life (although not a clinical term, I believe this is called high functioning ADHD) and can excel in areas that interest them but fail in others.*

Textually, the impression one can produce is that the person uses a mental-process verb with first-person subject, framing the description as the clinician’s perspective as perceived by the patient. This creates epistemic distance because the phrase should be filled with adds deontic force, attributing a specific threshold model to the clinician’s reasoning. The parenthetical insertion although not a clinical term it can be called high functioning ADHD performs stance work. Although not a clinical term, it acknowledges uncertain nosological status and this act can be labeled as an epistemic hedge. It employs a passive construction that deletes the agent, suggesting community language without claiming personal authorship.

A second exemplar from a woman in her thirties provides dense technical nominalization:

*Chronic inattention, executive dysfunction, task paralysis, time blindness, memory lapses, and emotional regulation challenges. These have been lifelong, predating any identified trauma and persisting across different life stages and contexts*.

Each term represents nominalization: chronic inattention (instead of I often cannot pay attention), executive dysfunction (instead of I struggle to organize tasks), task paralysis (instead of I cannot start things). The subsequent sentence directly addresses DSM-5 diagnostic requirements: lifelong establishes developmental trajectory; predating any identified trauma performs differential diagnosis; persisting across different life stages and contexts addresses pervasiveness.

At the discursive practice level, texts drew simultaneously on clinical-diagnostic discourse (DSM-5 criteria, technical terminology), community-consensus discourse (terms such as high functioning ADHD circulating in online communities), argumentative-forensic discourse (evidence marshaling, logical reasoning), and experiential-phenomenological discourse (first-person accounts). This produces a genre transformation of clinical case formulations, traditionally written by clinicians about patients, become self-written counter-case formulations (Gilmore et al., 2022).

### 4.5 Strategy 2: mobilization of biographical and pharmacological evidence (66.7%)

This strategy converted contested subjective experience into forms less open to dismissal: quantification, links to legal or financial consequences, documentary evidence, or medication-response patterns.

Exemplar:

*Just this year, I was facing over [GBP redacted] in fines from HMRC due to late filing of my self-assessments, a task I had been putting off due to a combination of task paralysis and time blindness.*

Textually, just this year establishes recency; over [GBP redacted] performs an evidential function through specificity; HMRC invokes state authority; fines (rather than charges) emphasizes punitive consequence. The causal construction creates a double chain: fines… due to late filing… a task I had been putting off due to task paralysis and time blindness, linking objective consequence to ADHD symptoms through the bridge of named impairment.

Pharmacological evidence appeared through medication-response arguments:

*At the end of the assessment, I asked why Ritalin would have had such a positive impact on my concentration, mental clarity and focus if I indeed did not have ADHD… ultimately, they could not provide an explanation*.

The rhetorical question presents an apparent contradiction that needs to be resolved. The reference to a positive impact describes benefits in several areas, including concentration, mental clarity, and focus. The phrase “ultimately, they could not provide an explanation” presents clinical authority as logically insufficient.

At the level of discursive practice, the patient uses an implied syllogism based on biomedical reasoning: ADHD medication treats specific deficits; Ritalin improved those deficits; therefore, those deficits must exist; therefore, ADHD must be the correct diagnosis. This reasoning contains several weak inferential steps, which are considered further in the Discussion. In particular, stimulant medications produce performance-enhancing effects on attention and executive function in non-ADHD populations (Smith and Farah, 2011; Volkow and Swanson, 2003), so a positive subjective response to Ritalin does not, in itself, constitute diagnostic evidence.

### 4.6 Strategy 3: hermeneutic reframing of clinical benevolence (98.0%)

This strategy treated the clinician’s interactional style (reassurance, normalization, mood exploration) as epistemically harmful because it allegedly disrupted appropriate information gathering, downplayed difficulties, or reframed neurodevelopmental distress as a mood problem.

Exemplar (under the patient’s own heading Misaligned assessor intentions):

*Throughout the session, the assessor kept focusing on my mood and my perception of my circumstances in an effort to ‘cheer me up’ and convince me that what I was experiencing wasn’t that bad. Whilst I believe their intention was good, it detracted from the very reason I was there.*

Textually, throughout the session signals persistent rather than incidental behavior; kept focusing uses the continuous form to highlight ongoing repetition; the quoted phrases ‘cheer me up’ and ‘wasn’t that bad’ use quotation marks as a distancing device, signaling contested utterances. The concessive Whilst I believe their intention was good, it detracted from… protects against charges of unfair characterization while asserting negative consequence regardless of intent; very and the very reason perform intensification.

### 4.7 Strategy 4: temporal conflict of masking (58.8%)

This strategy contrasted bounded assessment time with longer horizons during which impairment, exhaustion, or breakdown becomes visible. Masking or compensation was used to explain why outward performance during assessment constitutes invalid evidence against impairment.

Exemplar:

*The assessment report states I demonstrated ‘good eye contact’ and ‘appropriate social reciprocity’ during the clinical interview. However, this interpretation fails to recognize the exhausting compensatory strategies I employ in formal professional contexts. Maintaining eye contact requires conscious effort and causes significant distress which manifests hours later through emotional dysregulation and sensory overwhelm.*

Textually, the direct quotation of assessment report wording establishes the clinical observation as the starting point; quotation marks signal contestation; however introduces fundamental disagreement; this interpretation nominalizes the clinical observation into an evaluable entity; fails to recognize attributes epistemological limitation; exhausting compensatory strategies introduces compensation discourse; in formal professional contexts identifies the assessment as a high-stakes context eliciting peak performance. The temporal operator hours later is analytically central: it introduces a diachronic evidential model challenging the synchronic assessment snapshot, locating consequence outside the assessment’s temporal boundary.

A second exemplar documents environmental adaptation:

*When I went shopping at university, it would be 8.30 pm every night because it was quieter compared to midday. That was also the time they turned the music in the store off, so then all I had to deal with was smaller crowds and the temperature change between aisles, although the aisles still felt very cramped.*

Temporal precision (8.30 pm every night) establishes a systematic pattern; the diminutive all I had to deal with followed by an enumeration of further difficulties creates an ironic effect. Intertextual links to camouflaging and masking literature (Chapman, 2024; Lai et al., 2017) are evident, although these texts are not cited explicitly.

### 4.8 Structural-discursive tension T1: the competence paradox (86.3%)

Unlike S1 to S4, T1 is not a strategy used by patients but is a structural and discursive tension created by the format of the second opinion request.

We include it in the analytic scheme because it was highly common, appearing in 86.3% of cases, and is therefore theoretically important. However, we describe it as a tension produced by the institutional form itself, rather than as a deliberate strategy chosen by patients. Section “5 Discussion” considers the wider theoretical implications.

At the textual level, this tension can be seen in contradictions between what the patient says and the form in which they say it:

*I felt like my assessment was not very helpful as after speaking to other friends who have had assessments none of them had to do it via a video call. I find that I do not pay attention properly on video calls, I struggle to listen properly and fade out a bit unless I am actually face to face with somebody.*

Here, the patient describes difficulty paying attention during video calls and problems with listening. However, the request itself is a coherent written account. It requires attention, organization, and communicative ability to produce.

T1 becomes more marked as the request becomes longer. It was present in 93.3% of extensive requests, compared with 75.0% of brief requests. This suggests that longer requests create a stronger tension, because they require the sustained organization that the writer is, at the same time, claiming to lack.

## 5 Discussion

The Discussion is organized around three main theoretical points. First, we interpret the four strategies and the one structural-discursive tension using the framework of epistemic injustice. Second, we develop the concept of procedural epistemic injustice as the paper’s main theoretical contribution and distinguish it from related concepts. Third, we place the demographic and authorship patterns found in the corpus within their wider social and institutional context.

### 5.1 Three forms of epistemic injustice in neurodevelopmental assessment

Testimonial injustice (Carel and Kidd, 2014; Fricker, 2007) operates through the systematic discounting of credibility which is patients’ own knowledge of their cognitive and sensory functioning is treated as less reliable than what clinicians observe in short assessment sessions. Strategy 2 (mobilization of biographical and pharmacological evidence) is intelligible as a rational response to testimonial discounting; first-person accounts alone are deemed insufficient, so patients translate them into HMRC fines, medication responses, and other materially scaffolded warrants. The differential prevalence by condition (S2 used in 72.7% of ADHD versus 55.6% of autism requests) is consistent with differential availability of pharmacological warrant in ADHD pathways.

Women face specific testimonial injustice as their ADHD and autism presentations are routinely attributed to anxiety, personality, or attention-seeking (Gallagher et al., 2021). High-functioning individuals face a structurally analogous success paradox in which professional achievement is taken as evidence against impairment rather than as a product of effortful compensation (Lai et al., 2017; Loomes et al., 2017; Young et al., 2020). Both manifestations appear textually in S1 and S4.

Hermeneutical injustice arises when shared interpretive resources are insufficient to render an experience intelligible, leaving the speaker without conceptual purchase on their own situation (Carel and Kidd, 2014; Crichton et al., 2017; Fricker, 2007; Wardrope, 2015). In neurodevelopmental contexts, clinical frameworks remain dominated by criterion-based nosology (DSM-5, ICD-11) and a relatively narrow lexicon for the lived phenomenology of executive dysfunction, sensory regulation, and effortful social compensation (Chapman, 2024; Gallagher et al., 2021). Patients’ appropriation of community-generated terminology (time blindness, task paralysis, autistic burnout) is intelligible as a collective hermeneutical resource that fills these conceptual gaps (Chapman, 2024). Strategy 1 evidences this directly through the hedged use of community-originated terms (although not a clinical term, I believe this is called…). The same dynamic also speaks to the structurally analogous bind faced by women, whose presentations may be hermeneutically unintelligible to assessors trained on male-prototypical patterns (Loomes et al., 2017; Young et al., 2020).

Procedural epistemic injustice is the form to which we devote section “5.2 Procedural epistemic injustice: a distinct form.”

### 5.2 Procedural epistemic injustice: a distinct form

We argue that the competence paradox, T1, is not best understood as a patient strategy. It is better understood as evidence of a distinct form of epistemic injustice, which we call procedural epistemic injustice. This is the paper’s main theoretical contribution, and it needs to be distinguished from related ideas.

Procedural epistemic injustice is not the same as access injustice. Access injustice concerns whether a person can make a challenge at all, for example because of waiting lists, fees, language barriers, or physical access problems. The patients in our corpus were able to submit a challenge. The injustice occurs at the next stage.

It is also not the same as Fricker’s testimonial injustice. Testimonial injustice concerns whether a speaker is believed after they have spoken (Fricker, 2007). Our argument does not depend on whether the patient is believed or disbelieved. The procedural problem already exists before credibility is assessed.

This is also not the same as Fricker’s hermeneutical injustice because hermeneutical injustice concerns whether people have the shared concepts needed to make their experiences understandable (Fricker, 2007). In our corpus, patients often did make their experiences understandable, sometimes in highly developed ways. The problem is different and it concerns whether the institutional format for making a challenge excludes people whose impairment makes it difficult or impossible to produce a genre-competent written request.

Procedural epistemic injustice is also different from Dotson’s contributory injustice. Contributory injustice takes place when a listener fails to use available interpretive resources and therefore fails to understand the speaker (Dotson, 2011). Procedural epistemic injustice does not require willful neglect by any listener. It can operate through impersonal procedural and genre requirements. It can even operate before anyone reads the request, because the person may never have been able to produce the required text in the first place.

The main claim made here is that the format for making a challenge of the nature we are interested requires coherent prose, sustained attention, engagement with a multi-page document, alignment with diagnostic criteria, evidence-based reasoning, and anticipation of objections. These requirements may systematically exclude people whose impairment prevents them from meeting those expectations. At the same time, those who can meet the expectations may find that their competence is treated as evidence against the impairment they are claiming.

Two theoretical ideas help explain this process. First, Bourdieu’s concept of linguistic capital describes how socially valued ways of speaking and writing are unequally distributed (Bourdieu, 1991). The second opinion request requires a demanding form of linguistic capital using formal language, technical vocabulary, diagnostic alignment, and sustained narrative coherence. These skills are not equally available to all patients and the disadvantage is compounded because the conditions being challenged may themselves involve executive dysfunction, attentional impairment, and language-processing difficulties.

Goffman’s work on stigma management helps explain the second mechanism (Goffman, 1963). A patient requesting a second opinion has to perform two difficult tasks at once. They must show enough impairment to support their diagnostic claim, but also enough competence, organization, and credibility to be taken seriously. This creates a trap because to be accepted as a credible challenger, the patient must show competence. To be accepted as impaired, the patient must show difficulty or limitation. The genre therefore places the patient between two demands that are difficult to satisfy at the same time.

Procedural epistemic injustice has three defining features. First, it is structural rather than personal. It does not require any individual clinician, administrator, or institution to act wrongly. Second, it is often invisible because it appears as ordinary administrative process rather than as a barrier. Third, it is circular and self-defeating. More capable patients may be viewed with suspicion because their competence appears to weaken their claim, while more impaired patients may be unable to submit a challenge at all. In our corpus, T1, the competence paradox, is the visible sign of this process. Section “6 Operationalized recommendations” considers the implications for service design.

### 5.3 Power, medical Authority, and resource-driven gatekeeping

The findings point toward diagnostic authority as actively maintained through linguistic and institutional practices at least as far as it is perceived by patients rather than as naturally inhering in professional expertise. Clinical authority rests partly on knowledge and experience, but also on institutional positioning enabling authoritative knowledge claims whilst constraining patient challenges. Patients cannot simply assert diagnostic decisions but they must provide justifications, marshal evidence, anticipate objections, and demonstrate criterion knowledge. Clinicians face no equivalent burden as professional judgment has presumptive validity requiring no justification unless explicitly challenged.

Epistemic hierarchies privilege particular knowledge forms whilst devaluing others. Medical-scientific knowledge (biological mechanisms, quantified outcomes, standardized assessments) carries authority; experiential knowledge remains suspect. S2 reflects patients’ recognition of these hierarchies: phenomenological accounts alone are deemed insufficient, requiring translation into medical discourse or external validation.

S3, the hermeneutic reframing of clinical benevolence, contests this authority by recontextualizing clinical behaviors intended within a therapeutic discourse framework (reassurance, normalization, rapport-building) within an assessment validity discourse framework, arguing that such focus detracts from neurodevelopmental criteria. The reframing challenges not clinician malice but competing competence models: what clinicians view as holistic assessment is reframed as inappropriate deflection.

Resource scarcity can make diagnostic gatekeeping stricter than clinical judgment alone would require. NHS adult neurodevelopmental services work under major capacity pressures. In this context, higher diagnostic thresholds may reduce the number of people who meet criteria and therefore help manage demand. Patients’ apparent functioning, professional success, or use of compensation strategies may then be treated as evidence against diagnosis.

This means that diagnostic decision making may, in part, operate as an implicit rationing mechanism, with thresholds influenced by available resources as well as clinical criteria. We do not argue that this happened here but raise the possibility that it might in such cases and the literature on rationing through clinical judgment supports this as a plausible concern (Light, 2003).

S4, the temporal conflict of masking, addresses this problem as it challenges assessments that focus too narrowly on one point in time. S4 also varied strongly by author type. Professional advocates used it in all cases, whereas patients writing alone used it in 42.9% of cases. This pattern is itself relevant to procedural epistemic injustice. Explaining the long-term costs of compensation requires the ability to present evidence across time. Not all patients have equal access to that form of discourse competence.

### 5.4 Gender, Masking, and intersecting mechanisms of inequality

Women constituted 68.6% of the corpus despite ADHD and autism having been historically conceptualized as predominantly male conditions, and three-quarters of female requesters sought ADHD clarification specifically. The gender-condition association did not reach statistical significance in our underpowered subgroup analyses (Cramer’s *V* = 0.20), so we treat the pattern as suggestive rather than confirmatory.

Several intersecting mechanisms may contribute to this. First, recent meta-analyses suggest substantial female underdiagnosis in autism (Loomes et al., 2017) and consensus statements document under-recognition of ADHD in women (Young et al., 2020), supporting the interpretation that the female concentration partly reflects diagnostic inequality. Second, women may be more willing to engage formal healthcare challenge procedures, reflecting broader patterns of healthcare engagement. Third, women may experience greater identity-validation and credibility-restoration stakes that increase the perceived value of a successful challenge. Fourth, women may navigate bureaucratic procedures more readily than men, who may respond to negative outcomes with disengagement or by seeking private assessment. These explanations are not mutually exclusive.

The 68.6% figure may underestimate diagnostic inequality because of selection effects. The women able to submit detailed written challenges are likely to represent a more advantaged subgroup, with higher levels of educational, linguistic, and cultural capital. Women who are most affected by diagnostic inequality, including those with fewer resources or additional forms of disadvantage, may not appear in formal challenge procedures at all. Their absence is therefore not evidence that inequality is limited but instead, it may show procedural epistemic injustice operating in a gendered way.

### 5.5 Internet-mediated knowledge transformation and digital divides

The findings show significant transformation in patient-clinician knowledge asymmetries. Patients arrive at assessment having researched diagnostic criteria, read research literature, and engaged with online communities (Gilmore et al., 2022). Online neurodevelopmental communities provide education, generate collective knowledge, develop alternative terminology, and supply strategic knowledge about navigating healthcare systems. The high strategic density (76.5% of requests deploying three or more strategies) suggests shared rhetorical templates circulating through community networks.

Community networks can give patients access to shared knowledge, language, and interpretive resources.

However, the democratization of knowledge also creates new power dynamics (Caputo and Cervino, 2025). Clinicians may respond defensively when patients present challenges, seeing them as questioning professional expertise or engaging in cyberchondria (Starcevic and Berle, 2013). Diagnostic thresholds may then rise in response. When patients show sophisticated self-knowledge, this may be interpreted as evidence of capability rather than impairment. This strengthens T1.

Internet access also creates new inequalities because people with higher education, stronger digital skills, good English proficiency, and more time to research are better placed to produce detailed challenges. The difference between short and long requests supports this. Longer submissions contained more biographical evidence and showed T1 more often. Procedural injustice therefore operates along a gradient of digital and linguistic capital.

### 5.6 Standardized and neuropsychological assessment in contested cases

If digital and linguistic capital shape which patients can challenge, the kind of evidence a challenge can invoke is shaped in turn by what standardized and neuropsychological testing can and cannot establish. Self-administered screening instruments such as the AQ-50 and the RAADS, together with ADHD rating scales, are designed for case detection rather than confirmation and they are calibrated for sensitivity at the cost of specificity, so that a high score raises the prior probability of a condition without establishing it. In the corpus this distinction is important, because the single requester who went back and completed screening tools and then presents them as objective proof of an outcome the assessment had not reached it is treating a screen as though it carried confirmatory weight it was never intended to have. Neuropsychological testing of attention, working memory, or executive function is, on current evidence, corroborative rather than determinative so performance within normal limits does not exclude a neurodevelopmental condition, and impaired performance is not specific to one. As they stand their test results can only inform but cannot settle a criterion-based judgment.

The value of evidence of such instruments in contested cases is consequently real but bounded and cannot alone be used as a tool to determine the outcome. They are most useful where their limits are stated for example a screen can justify proceeding to assessment, a standardized observation can document what was and was not seen, and a neuropsychological profile can add or qualify a strand of evidence, but none substitutes for the longitudinal, criterion-referenced clinical formulation that distinguishes those who meet diagnostic criteria from those who do not.

On this sample of challenged testing outcomes what requesters contested was the interpretation of behavior recorded during the assessment, reframing observations such as good eye contact and appropriate social reciprocity as artifacts of effortful compensation (S4, 58.8%), and the conduct of the interview itself (S3, 98.0%). Where standardized testing entered at all, it did so as screening evidence was offered afterward in support of the challenge. This pattern is itself a finding as in this service and this corpus, contested neurodevelopmental decisions turn on the interpretation of observed and reported behavior as recorded by clinicians against criteria, not on psychometric outputs, which suggests that expanding the place of standardized testing would shift rather than resolve the locus of disagreement to another area of challenge.

## 6 Operationalized recommendations

Our findings lend themselves to six operational recommendations for improving the assessment pathway and reducing the likelihood that relevant evidence only emerges after an unfavorable diagnostic decision. Table 4 draws the analysis together before these recommendations are set out: for each finding it states the form of epistemic injustice involved, the safeguard the finding implies, and the question it leaves open for research. The safeguards follow analytically from the findings, but their effects on diagnostic accuracy, demand, and equity are themselves empirical questions, set out in the final column.

**TABLE 4:** Synthesis of discursive findings, forms of epistemic injustice, proposed safeguards, and future directions.

Discursive finding (prevalence)	Form of epistemic injustice	Proposed service-level safeguard	Future research direction
Appropriation and recalibration of clinical taxonomy (98.0%)	Hermeneutical: clinical vocabulary inadequate to lived experience	Test community-originated descriptors on substance against criteria; state which criteria are engaged	Whether structured recognition of compensated presentations changes outcomes without reducing specificity
Mobilization of biographical and pharmacological evidence (66.7%)	Testimonial: phenomenological accounts discounted relative to objective proxies	Declare in advance which forms of evidence a reconsideration weighs and how	Incremental validity of self-reported consequences and screening scores over criterion-based judgment
Hermeneutic reframing of clinical benevolence (98.0%)	Testimonial and procedural: interpretive authority over the encounter held asymmetrically	Record how each reported difficulty was interpreted and why; make credibility judgments reviewable	Whether transparent reasoning reduces reconsideration rates and grievance
Temporal conflict of masking (58.8%; 100% in advocate-authored requests)	Testimonial: cross-sectional observation privileged over longitudinal report	Specify how longitudinal and collateral information is weighed; offer supported or oral accounts	Sustainability and diagnostic significance of compensation, given contested camouflaging evidence
Competence paradox (T1; 86.3%, 93.3% in extensive requests)	Procedural: genre demands the capacities the conditions impair	Exclude the sophistication of a submission from the evidential basis; provide scaffolding and advocacy as of right	Whether procedural support alters which requesters challenge and with what outcome

### 6.1 Pre-assessment evidence schedule

First, an extended assessment should not mean simply making appointments longer but it should mean redesigning the early assessment pathway so that the issues most often raised at second opinion stage are explored before the initial decision is made. In practice, this would involve a pre-assessment evidence schedule, completed before the clinical interview, asking patients to give concrete examples of impairment across education, employment, domestic life, relationships, finances, sensory load, emotional regulation, and administrative functioning. The schedule should ask not only whether a difficulty exists, but how the person manages it, what compensatory strategies are used, what those strategies cost, and what happens when they break down. This would bring evidence currently produced retrospectively in second opinion requests into the initial assessment itself, reducing the need for patients to construct a counter-case after an unfavorable outcome.

### 6.2 Dedicated assessment of masking and compensation

Second, masking and compensation strategies should be assessed as a clinical domain in their own right, rather than treated as a later explanation for apparent discrepancies. Initial assessment templates should therefore include a dedicated masking section. This could examine performance during formal appointments, functioning in work or education, post-event exhaustion, delayed emotional dysregulation, sensory overload after apparently successful interaction, and context-dependent decompensation. The main question to address is not whether the person appeared socially competent or organized during the appointment, but whether that presentation was effortless, sustainable, and representative of everyday functioning. This would address the temporal conflict identified in the corpus, where patients challenged the evidential weight given to a single, time-limited assessment encounter.

### 6.3 Targeted assessor training

Third, assessor training should be made more specific and should include worked examples showing how women, high-achieving individuals, and people with strong verbal ability may still experience clinically significant impairment despite superficially intact functioning. Training should also address the limits of medication-response reasoning, the clinical status of community-derived terms such as time blindness, task paralysis, autistic burnout, and high-functioning ADHD, and the risk of prematurely attributing neurodevelopmental presentations to anxiety, depression, personality, or ordinary stress. These alternatives should be considered, but only after developmental onset, pervasiveness, impairment, and differential explanations have been properly tested and found not to feature in the diagnosis.

### 6.4 Transparent diagnostic documentation

Fourth, diagnostic assessments need to be strengthened at the level of documentation. Reports should show explicitly how each major self-reported difficulty was considered, what evidence supported or weakened it, how alternative explanations were weighed, and why the diagnostic threshold was or was not met. Where functioning is used as evidence against diagnosis, the report should distinguish between genuine absence of impairment and impairment reduced or hidden by compensation. Where anxiety, trauma, depression, substance use, or personality-related explanations are judged more plausible, the report should explain why they better account for the presentation, rather than simply listing them as alternatives. This would make clinical reasoning more transparent and reduce the risk that reassurance, normalization, or mood exploration is experienced as diagnostic deflection.

### 6.5 Alternative routes for reconsideration

Fifth, services should avoid relying only on formal written second opinion requests as the main route for challenging an assessment outcome. The competence paradox identified in this study suggests that the ability to produce a coherent, evidence-rich written challenge may itself be interpreted as counter-evidence, while those with greater executive, linguistic, or attentional impairment may be least able to challenge the decision at all. A fairer model would offer alternative routes, such as a structured reconsideration form, a brief supported feedback appointment, collateral evidence from relatives or workplace sources, and a defined process for submitting missing developmental or functional information before a formal second opinion threshold is reached. These mechanisms would not lower diagnostic standards. They would improve the quality and fairness of the evidence available at the first decision point.

### 6.6 An equity-oriented model for reconsideration

Sixth, the reconsideration process itself should be governed by three principles that can be stated without resolving the resource tensions the analysis identifies. The first is parity of sources of information. Because the corpus shows requesters converting subjective experience into fiscal, legal, and pharmacological lines of argument precisely because phenomenological accounts are discounted (the biographical strategy, 66.7%), a fairer process would specify in advance which forms of evidence a reconsideration for second opinion will weigh and how, rather than leaving testimony of their experience to compete on unequal terms with whatever objective third party information a requester happens to be able to assemble. Stating the evidential standard openly would remove the hidden premium currently placed on the social and educational ability required to produce quantified consequences. The second principle is independence of decision. Where the same service, and ideally a clinician not involved in the original assessment, reviews its own contested decision, the reviewer inherits the institutional interest in the original outcome; structural separation of the reconsideration from the original decision is the minimal safeguard against the threshold increase the analysis attributes to demand management. The third principle is proportionate scaffolding. Because the competence paradox (86.3%, rising to 93.3% in extensive requests) shows that the genre itself selects for the very capacities the contested conditions affect, equity requires that the burden of mounting a challenge be reduced for those least able to meet it, through the alternative routes set out in 6.5 for example structured prompts, the option of an oral or supported account, and advocacy assistance offered as of right rather than discovered by the resourceful.

These principles address the specific biases the corpus makes visible. Reducing testimonial injustice requires that a reconsideration record, in terms a requester can see, how each self-reported difficulty was interpreted and why it was found to meet or not meet a criterion, so that credibility judgments become reviewable rather than implicit. Reducing procedural injustice requires that the sophistication of a submission be formally excluded from the evidential basis of the decision. The analysis documents responses treating the quality of a written challenge as evidence of preserved executive function, and a fair process must direct reviewers that the manner of a challenge carries no diagnostic weight. Reducing hermeneutical injustice requires that community-originated descriptors such as time blindness or task paralysis be heard as attempts to name experience for which clinical vocabulary is thin, and tested against criteria on their substance, rather than dismissed on their provenance.

It should be stated however that fairness and diagnostic rigor are complementary rather than opposed, because the natural objection to each of these safeguards is that they invite diagnostic inflation. The safeguard against inflation is not procedural obstruction but auditable and clear clinical reasoning. A process that requires the reviewer to show their working by stating which criteria are engaged, what evidence bears on each, and why the threshold is or is not met, disciplines both over-diagnosis and under-diagnosis, because both become visible as departures from a stated standard. Rigor is degraded, not protected, by opacity, since an unexplained refusal is indistinguishable from an arbitrary one and generates precisely the epistemic grievance the corpus records. Equity-oriented reconsideration and criterion-based discipline therefore point in the same direction that a decision which can be explained is both fairer to the requester and more defensible as clinical science.

Taken together, these changes would move services away from retrospective dispute management and toward prospective epistemic design. The purpose is not to accept every contested diagnostic claim, or to treat subjective conviction as diagnostic proof. It is to ensure that the initial assessment actively examines the domains that later become sources of dispute: masking, compensation, functional cost, biographical impairment, medication narratives, and the patient’s understanding of the clinical reasoning. This makes the recommendations actionable by specifying what should happen, when it should happen, who should do it, and how it should be built into the assessment pathway. None of this will dissolve the resource constraint the analysis foregrounds. Independent review, scaffolding, and auditable reasoning all consume clinician time that capacity-limited services do not have, and a more accessible challenge route may raise demand that the same services cannot meet. The recommendation is therefore not that these safeguards are costless, but that their cost should be made explicit and owned at the level of commissioning, rather than displaced onto individual threshold judgments where it currently operates as invisible rationing.

## 7 Limitations

Several limitations qualify our findings.

### 7.1 Sample generalizability and intersectional gaps

The corpus of 51 second opinion requests was drawn from a single NHS adult neurodevelopmental service. The overrepresentation of self-referred women (68.6%) suggests a specific subgroup possessing the literacy, tenacity, and institutional knowledge to navigate formal challenge procedures. This excludes the experiences of those most marginalized by diagnostic systems, including individuals who, owing to severity of impairment, lack of advocacy, digital exclusion, or systemic distrust, disengage or cannot formulate a written appeal. The absence of demographic data on race, ethnicity, socioeconomic status, and co-occurring disabilities precludes an intersectional analysis.

### 7.2 Single-coder constraint

All coding was performed by one researcher (MA). We addressed this through the trustworthiness framework described in section “3.7 Trustworthiness in place of inter-rater reliability” (analytic memos, conservative coding rule, negative case analysis, peer debriefing within the interdisciplinary team, audit trail, exemplar quotation density). We make no claim that these procedures fully substitute for inter-rater reliability quantification and recommend that future studies recruit a second coder for at least a 20% subsample with Cohen’s kappa reported.

### 7.3 Statistical underpower

Inferential tests on the self-referred subgroup (*n* = 39) were severely underpowered, with minimum detectable effects of d approximately 1.0. Non-significant results should not be interpreted as evidence of absence. We have reported effect sizes with confidence intervals to permit independent assessment of the data’s substantive implications, but the inferential framing carries limited weight.

### 7.4 Focus on formal written appeals

By design, the analysis captured only one form of diagnostic dispute. Informal complaints, private assessments, and service disengagement are not analysed and may represent the predominant experiences of many.

### 7.5 Analytic neutrality and clinical accuracy

The analysis does not adjudicate the clinical accuracy of the original diagnoses or the second opinion requests. We treat patient distress and epistemic claims as legitimate social facts for analysis, not as clinical truths to be verified. The clinical validity of patient critiques remains an important separate empirical question, and prior work has shown that the relationship between subjective complaint, observable functioning, and confirmed diagnostic status in adult ADHD and autism is neither linear nor unidirectional (Mahdi et al., 2018).

### 7.6 Researcher positionality

Our interdisciplinary team lacked dedicated expertise in critical social theory, disability studies, and intersectionality. Future collaborative work should include scholars with these specializations.

## 8 Future research

Future research should diversify samples across multiple sites and actively recruit participants from marginalized communities, adopting an intersectional lens to examine how race, class, disability, and other axes of inequality shape diagnostic experiences and challenge outcomes. Inclusive methods such as oral histories and plain-language surveys would permit participation by digitally excluded or literacy-challenged patients. Collaborations with disability studies scholars, critical race theorists, and social scientists would deepen the analysis of power dynamics. Quasi-experimental evaluation of the operationalized recommendations in section “6 Operationalized recommendations” is a priority. Empirical study of the alternative challenge formats proposed in section “6.3 Targeted assessor training” would test whether procedural epistemic injustice can be measurably reduced.

## 9 Conclusion

This critical discourse analysis of second opinion requests reveals that disputes over neurodevelopmental diagnosis constitute fundamental epistemic conflict, rooted in competing frameworks of evidence, authority, and experience. By examining the architecture of these appeals, we have charted how patients navigate and contest the power dynamics of clinical judgment, transforming subjective distress into structured institutional arguments.

The analysis makes three main contributions. First, it documents four patient strategies (appropriation and recalibration of clinical taxonomy, mobilization of biographical and pharmacological evidence, hermeneutic reframing of clinical benevolence, temporal conflict of masking) that together represent a direct challenge to the rules governing what counts as valid knowledge within the diagnostic assessment. Second, it argues that the competence paradox is not something patients deliberately create, but a structural and discursive trap built into the genre itself. It treats this paradox as the characteristic textual marker of a specific kind of epistemic injustice, which it terms procedural. Third, it distinguishes procedural epistemic injustice from access barriers, from Fricker’s testimonial and hermeneutical pair, and from Dotson’s contributory injustice, and connects it to Bourdieu’s linguistic capital and Goffman’s stigma management to give it theoretical depth.

Methodologically, the study underscores the value of applying CDA to frontline institutional genres. Second opinion requests provide a window into reasoning-in-action, capturing the tactical labor of reconstructing a contested identity within strict bureaucratic and epistemic confines.

The conflicts seen in these documents are signs of a system under serious pressure. We believe they arise from two main tensions. The first is the gap between the ideal of detailed, longitudinal assessment and the reality of limited, time-pressured services. The second is the gap between an older model of the passive patient and the reality of the informed, connected patient who uses clinical, community, and online knowledge to challenge decisions.

Addressing these epistemic injustices therefore requires system-level reform, not just changes in individual clinician behavior. This should include clearer assessment protocols, better clinician training, improved design of challenge procedures, and greater transparency about diagnostic thresholds.

In taking these appeals seriously as sites of epistemic production, the analysis reframes the question. The salient inquiry is no longer whether individual patients are correct, but what the pervasive form and persuasive force of their challenges reveal about the system that produces them.

## Data Availability

The data analyzed in this study can be made available on request. Requests to access these datasets should be directed to MA, m.adamou@hud.ac.uk.

## References

[B1] Adamou M. Arif M. Asherson P. Cubbin S. Leaver L. Sedgwick-Müller J. et al. (2024). The adult ADHD assessment quality assurance standard. *Front. Psychiatry* 15:1380410. 10.3389/fpsyt.2024.1380410 39156609 PMC11327143

[B2] Ainsworth-Vaughn N. (1998). *Claiming Power in Doctor-Patient Talk.* Oxford: Oxford University Press.

[B3] American Psychiatric Association. (2013). *Diagnostic and Statistical Manual of Mental Disorders*, 5th Edn. Washington, DC: American Psychiatric Association, 10.1176/appi.books.9780890425596

[B4] Bourdieu P. (1991). *Language and Symbolic Power.* Cambridge: Polity Press.

[B5] Braun V. Clarke V. (2023). Is thematic analysis used well in health psychology? A critical review of published research, with recommendations for quality practice and reporting. *Health Psychol. Rev.* 17 695–718. 10.1080/17437199.2022.2161594 36656762

[B6] Candlin C. N. Candlin S. (2002). Introduction: Discourse, expertise, and the management of risk in health care settings. *Res. Lang. Soc. Interaction* 35 115–137. 10.1207/S15327973RLSI3502_1 42418030

[B7] Caputo F. Cervino C. (2025). “The drama of healthcare democratisation in the digital era,” in *Digital Tools and Data for Innovative Healthcare*, eds de Pablos P. O. Lytras M. D. (London: Academic Press), 279–298.

[B8] Carel H. Kidd I. J. (2014). Epistemic injustice in healthcare: A philosophical analysis. *Med. Health Care Philosophy* 17 529–540. 10.1007/s11019-014-9560-2 24740808

[B9] Chapman R. (2024). *Empire of Normality: Neurodiversity and Capitalism.* London: Pluto Press.

[B10] Cohen J. (1988). *Statistical Power Analysis for the Behavioral Sciences*, 2nd Edn. Hillsdale, NJ: Lawrence Erlbaum Associates.

[B11] Crichton P. Carel H. Kidd I. J. (2017). Epistemic injustice in psychiatry. *BJPsych Bull.* 41 65–70. 10.1192/pb.bp.115.050682 28400962 PMC5376720

[B12] Cruz S. Zubizarreta S. C. Costa A. D. Araújo R. Martinho J. Tubío-Fungueiriño M. et al. (2025). Is there a bias towards males in the diagnosis of autism? A systematic review and meta-analysis. *Neuropsychol. Rev.* 35 153–176. 10.1007/s11065-023-09630-2 38285291 PMC11965184

[B13] Dotson K. (2011). Tracking epistemic violence, tracking practices of silencing. *Hypatia* 26 236–257. 10.1111/j.1527-2001.2011.01177.x

[B14] Fairclough N. (1992). *Discourse and Social Change.* Cambridge: Polity Press.

[B15] Fairclough N. (2013). *Critical Discourse Analysis: The Critical Study of Language*, 2nd Edn. London: Routledge.

[B16] Fricker M. (2007). *Epistemic Injustice: Power and the Ethics of Knowing.* Oxford: Oxford University Press.

[B17] Gallagher S. Little J. M. Hooker C. (2021). Testimonial injustice: Discounting women’s voices in health care priority setting. *J. Med. Ethics* 47 744–747. 10.1136/medethics-2019-105984 32332153

[B18] Gilmore R. Beezhold J. Selwyn V. Howard R. Bartolome I. Henderson N. (2022). Is TikTok increasing the number of self-diagnoses of ADHD in young people? *Eur. Psychiatry* 65:S571. 10.1192/j.eurpsy.2022.1463

[B19] Goffman E. (1963). *Stigma: Notes on the Management of Spoiled Identity.* Englewood Cliffs, NJ: Prentice-Hall.

[B20] Iedema R. (2007). *The Discourse of Hospital Communication: Tracing Complexities in Contemporary Health Care Organizations.* Basingstoke: Palgrave Macmillan.

[B21] Knott R. Mellahn O. J. Tiego J. Kallady K. Brown L. E. Coghill D. et al. (2024). Age at diagnosis and diagnostic delay across attention-deficit hyperactivity and autism spectrums. *Australian N. Z. J. Psychiatry* 58 142–151. 10.1177/00048674231206997 37885260 PMC10838471

[B22] Kraemer H. C. (2007). DSM categories and dimensions in clinical and research contexts. *Int. J. Methods Psychiatric Res.* 16 S8–S15. 10.1002/mpr.211 17623398 PMC6879071

[B23] Kurs R. Grinshpoon A. (2018). Vulnerability of individuals with mental disorders to epistemic injustice in both clinical and social domains. *Ethics Behav.* 28 336–346. 10.1080/10508422.2017.1365302

[B24] Lai M. C. Lombardo M. V. Ruigrok A. N. Chakrabarti B. Auyeung B. Szatmari P. et al. (2017). Quantifying and exploring camouflaging in men and women with autism. *Autism* 21 690–702. 10.1177/1362361316671012 27899710 PMC5536256

[B25] Leach J. (2000). “Rhetorical analysis,” in *Qualitative Researching with Text, Image and Sound: A Practical Handbook*, eds Bauer M. W. Gaskell G. (London: Sage), 207–226.

[B26] Light D. W. (2003). Universal health care: Lessons from the British experience. *Am. J. Public Health* 93 25–30. 10.2105/AJPH.93.1.25 12511379 PMC1447686

[B27] Lincoln Y. S. Guba E. G. (1985). *Naturalistic Inquiry.* Beverly Hills, CA: Sage.

[B28] Loomes R. Hull L. Mandy W. P. (2017). What is the male-to-female ratio in autism spectrum disorder? A systematic review and meta-analysis. *J. Am. Acad. Child Adolesc. Psychiatry* 56 466–474. 10.1016/j.jaac.2017.03.013 28545751

[B29] Mahdi S. Viljoen M. Massuti R. Selb M. Almodayfer O. Karande S. et al. (2018). An international qualitative study of functioning in autism spectrum disorder using the World Health organization international classification of functioning, disability and health framework. *Autism Res.* 11 463–475. 10.1002/aur.1905 29226604 PMC5900830

[B30] National Institute for Health and Care Excellence. (2012). *Autism Spectrum Disorder in Adults: Diagnosis and Management. (NICE Clinical Guideline CG142).* London: NICE. 32186834

[B31] National Institute for Health and Care Excellence. (2018). *Attention Deficit Hyperactivity Disorder: Diagnosis and Management. (NICE Guideline NG87).* London: NICE. 29634174

[B32] NHS Digital. (2024). *Autism Statistics, April 2023 to March 2024.* London: NHS Digital.

[B33] Nowell L. S. Norris J. M. White D. E. Moules N. J. (2017). Thematic analysis: Striving to meet the trustworthiness criteria. *Int. J. Qual. Methods* 16 1–13. 10.1177/1609406917733847

[B34] Putnam L. L. Grant D. Michelson G. Cutcher L. (2005). Discourse and resistance: Targets, practices, and consequences. *Manag. Commun. Quart.* 19 5–18. 10.1177/0893318905276557

[B35] Sarangi S. Roberts C. (1999). *Talk, Work and Institutional Order: Discourse in Medical, Mediation and Management Settings.* Berlin: Mouton de Gruyter.

[B36] Smith M. C. Mukherjee R. A. Müller-Sedgwick U. Hank D. Carpenter P. Adamou M. (2024). UK adult ADHD services in crisis. *BJPsych Bull.* 48 1–5. 10.1192/bjb.2023.88 38058161 PMC10801359

[B37] Smith M. E. Farah M. J. (2011). Are prescription stimulants “smart pills”? The epidemiology and cognitive neuroscience of prescription stimulant use by normal healthy individuals. *Psychol. Bull.* 137 717–741. 10.1037/a0023825 21859174 PMC3591814

[B38] Srivarathan A. Bradford A. Shearkhani S. Heimlich L. Jefferson S. Miller K. E. et al. (2025). Bridging diagnostic safety and mental health: A systematic review highlighting inequities in autism spectrum disorder diagnosis. *BMJ Quality Saf* 10.1136/bmjqs-2025-018723 Advance online publication. 40854799 PMC12707049

[B39] Starcevic V. Berle D. (2013). Cyberchondria: towards a better understanding of excessive health-related Internet use. *Expert Rev. Neurotherapeutics* 13 205–213. 10.1586/ern.12.162 23368807

[B40] Volkow N. D. Swanson J. M. (2003). Variables that affect the clinical use and abuse of Methylphenidate in the treatment of ADHD. *Am. J. Psychiatry* 160 1909–1918. 10.1176/appi.ajp.160.11.1909 14594733

[B41] Wardrope A. (2015). Medicalization and epistemic injustice. *Med. Health Care Philosophy* 18 341–352. 10.1007/s11019-014-9608-3 25374423

[B42] Wodak R. (2009). *The discourse of Politics in Action: Politics as Usual.* Basingstoke: Palgrave Macmillan.

[B43] World Health Organization. (2019). *International Classification of Diseases*, 11th Edn. Geneva: World Health Organization.

[B44] Young S. Adamo N. Ásgeirsdóttir B. B. Branney P. Beckett M. Colley W. et al. (2020). Females with ADHD: An expert consensus statement taking a lifespan approach providing guidance for the identification and treatment of attention-deficit/hyperactivity disorder in girls and women. *BMC Psychiatry* 20:404. 10.1186/s12888-020-02707-9 32787804 PMC7422602

